# Homeostasis of cellular amino acids in *Acanthamoeba castellanii* exposed to different media under amoeba-bacteria coculture conditions

**DOI:** 10.1186/s12866-023-02942-6

**Published:** 2023-07-26

**Authors:** Chih-Ming Tsai, Chun-Hsien Chen, Wei-Hung Cheng, Foekje F. Stelma, Sung-Chou Li, Wei-Chen Lin

**Affiliations:** 1 Institute of Basic Medical Sciences, College of Medicine, National Cheng Kung University, Tainan, Taiwan; 2 Department of Parasitology, College of Medicine, National Cheng Kung University, Tainan, Taiwan; 3 Department of Microbiology and Immunology, College of Medicine, National Cheng Kung University, Tainan, Taiwan; 4 Department of Microbiology, Radboud University Nijmegen Medical Centre, Nijmegen, Netherlands; 5 Department of Medical Education and Research, Kaohsiung Veterans General Hospital, Kaohsiung, Taiwan; 6 Department of Nursing, Meiho University, Pingtung, Taiwan

**Keywords:** *Acanthamoeba castellanii*, Amino acids, Metabolomic, Coculture

## Abstract

**Background:**

*Acanthamoeba castellanii* is a free-living protist that feeds on diverse bacteria. *A. castellanii* has frequently been utilized in studies on microbial interactions. Grazing bacteria also exhibit diverse effects on the physiological characteristics of amoebae, such as their growth, encystation, and cytotoxicity. Since the composition of amoebae amino acids is closely related to cellular activities, it can indicate the overall responses of *A. castellanii* to various stimuli.

**Method:**

*A. castellanii* was exposed to different culture conditions in low-nutrient medium with heat-killed DH5α to clarify their effects. A targeted metabolomic technique was utilized to evaluate the concentration of cellular amino acids. The amino acid composition and pathways were analyzed by two web-based tools: MetaboAnalyst and Pathview. Then, long-term exposure to *A. castellanii* was investigated through in silico and in vitro methods to elucidate the homeostasis of amino acids and the growth of *A. castellanii.*

**Results:**

Under short-term exposure, all kinds of amino acids were enriched in all exposed groups. In contrast to the presence of heat-killed bacteria, the medium exhibited obvious effects on the amino acid composition of *A. castellanii*. After long-term exposure, the amino acid composition was more similar to that of the control group. *A. castellanii* may achieve amino acid homeostasis through pathways related to alanine, aspartate, citrulline, and serine.

**Discussion:**

Under short-term exposure, compared to the presence of bacteria, the type of medium exerted a more powerful effect on the amino acid composition of the amoeba. Previous studies focused on the interaction of the amoeba and bacteria with effective secretion systems and effectors. This may have caused the effects of low-nutrient environments to be overlooked.

**Conclusion:**

When *A. castellanii* was stimulated in the coculture system through various methods, such as the presence of bacteria and a low-nutrient environment, it accumulated intracellular amino acids within a short period. However, different stimulations correspond to different amino acid compositions. After long-term exposure, *A. castellanii* achieved an amino acid equilibrium by downregulating the biosynthesis of several amino acids.

**Supplementary Information:**

The online version contains supplementary material available at 10.1186/s12866-023-02942-6.

## Background

The free-living protist *Acanthamoeba castellanii* plays multiple roles in infectious diseases. For instance, the protist can opportunistically infect the human cornea and cause the sight-threatening corneal disease Acanthamoeba keratitis (AK). Recently, the incidence of AK has increased markedly [1, 2]. *A. castellanii* can attach to mammalian cells via mannose-binding protein and secrete aminopeptidases that cause cytopathic effects [3, 4]. *A. castellanii* can also induce virulence in environmental microbes, including bacteria, fungi, and viruses, through prey‒predator interactions [3, 5–7]. *A. castellanii* also provides intracellular niches for various bacteria and serves as a shelter, allowing these bacteria to avoid harsh environments [8–11]. On the other hand, the interaction between amoebae and bacteria influences the phenotype of *A. castellanii*, including its ability to grow, encystation, and cytotoxicity [12, 13]. However, many mechanisms underlying the amoeba-bacteria interaction remain unknown.

To investigate the amoeba-bacteria interaction, several amoeba-bacteria coculture experiments have been employed. The coculture systems currently used frequently involve nonnutrient saline, such as Page’s amoeba saline [14–16], or nutrient medium diluted with saline [17]. As both amoebae and bacteria overgrow in nutrient-rich medium, a non or low-nutrient medium is used in coculture experiments. Furthermore, some nonnutrient media, such as artificial seawater, are more similar to natural habitats [18]. However, this experimental design causes amoebae to be simultaneously exposed to a low-nutrient environment and bacteria, making it difficult to disentangle the effects of each factor. Additionally, due to the lack of molecular tools and related software, the cellular physiology of *A. castellanii* under varying coculture systems has been hard to analyze.

In recent years, metabolomic techniques have been widely used in biomarker identification. For example, previous studies utilized metabolomics to discriminate malignant pleural mesothelioma cell lines [19]. In protist studies, metabolomics has been used to characterize different life stages of *Toxoplasma gondii* [20]. Those studies found that cellular amino acids can serve as signatures for cell types or status. Cellular amino acids can regulate cell sensing pathways and modulate amino acid production, consumption, and temporal storage in organelles, such as lysosomes [21]. A previous study found that mammalian cells accumulate essential amino acids within the lysosome in response to short-term amino acid starvation [22]. Cellular amino acids not only affect amino acid metabolism but also play an essential role as basic metabolites as well as metabolic regulators in various pathways. Moreover, the composition and amount of cellular amino acids also indicate that some heterotrophic protists are adapted to hypersaline environments [23].

This study aims to analyze the intracellular amino acid metabolism of *A. castellanii* during bacterial grazing. Due to the difficulties in controlling bacterial growth, we used heat-killed *Escherichia coli* DH5α in our experiments. A targeted amino acid metabolomic approach was applied to investigate 22 amino acids and analyze the amino acid composition of each amoebic exposure group. The web-based tool MetaboAnalyst was used. After a 3-day short-term exposure to different compositions of media, all exposed groups of *A. castellanii* accumulated most of the amino acids measured. However, amino acids were not accumulated in the untreated control group. After a 21-day long-term exposure to coculture medium (low-nutrient medium with heat-killed *E. coli*), a more similar amino acid composition was observed with the experimental group than with the nonexposed control group.

To further investigate the regulation of amino acid metabolism, the web tool Pathview was used to integrate the results of metabolomics and RNA-seq for pathway analysis. The results demonstrated that after long-term exposure to the coculture media, *A. castellanii* may achieve amino acid homeostasis by regulating two pathways: the arginine biosynthesis pathway (KEGG map: 00220) and glycine-serine-threonine metabolism pathway (KEGG map: 00260). When considering the long-term exposure group, the growth curve showed two log phases, which might be related to (1) the initial bacterial grazing and (2) the subsequent acclimation in a low-nutrient environment. In this study, the homeostasis of cellular amino acids was examined, and the amino acid metabolism in bacterial grazing on *A. castellanii* was elucidated. We also found that the amoeba needs long-term incubation to acclimate to the low-nutrient environment. However, since the amoeba could constantly graze on bacteria and maintain the trophozoite morphology, its physiological state is often overlooked.

## Methods

### Culture of *A. castellanii* and *E. coli* DH5α

*A. castellanii* was cultured as described previously [24]. In brief, *A. castellanii* strain ATCC_30010 was cultured in protease peptone-yeast extract-glucose (PYG) medium (pH 6.5) at 28 °C in culture flasks. Trophozoites were harvested after cultivation for 3–5 days during the logarithmic growth phase. *E. coli* DH5α was inoculated to the logarithmic growth phase with OD = 1.0 (approximately 1.7 × 10^8^ CFU/mL) for subsequent processing or coculture experiments. Due to the difficulties in controlling bacterial growth in the coculture system, we used heat-killed *Escherichia coli* DH5α in the experiments.

### Medium exposure in coculture

To evaluate the intracellular amino acid composition and metabolic pathways in grazing amoebae, we compared *A. castellanii* ATCC_30010 exposure to different culture conditions, including medium compositions and exposure times. Several groups of amoeba with different exposures were created, including the following: (1) Control group, the normal cultured amoeba exposed to undiluted PYG medium; (2) Group A, amoeba exposed to PYG medium diluted to 20%; (3) Group B, amoeba exposed to undiluted PYG medium with heat-killed bacteria for 3 days; (4) Group C, amoeba exposed to PYG medium diluted to 20% with heat-killed bacteria for 3 days; and (5) Group E3, amoeba exposed to PYG medium diluted to 20% with heat-killed bacteria for more than 20 days. After exposure, the intracellular metabolites of the amoeba were extracted for metabolomic assays.

The 20% PYG medium was made by diluting 1 volume of PYG with 4 volumes of Page’s amoeba saline (PAS). To produce heat-killed *E. coli* DH5α, bacteria at OD = 1.0 were collected by centrifugation and suspended in the corresponding medium (100% or 20% PYG medium). Then, the suspended *E. coli* DH5α was heated at 100 °C for 10 min. The heat-killed bacteria were finally diluted to 2.0 × 10^6^ CFU/mL. A total of 100 µg/mL ampicillin was added to each medium.

### Bacterial grazing test

*E. coli* DH5α was incubated with different groups of *A. castellanii*. E3: *E. coli* DH5α cocultured with *Acanthamoeba* E3 group, m: only *E. coli* DH5α in medium, n: *E. coli* DH5α cocultured with normally cultured ATCC_30010, s: *E. coli* DH5α cocultured with ATCC_30010 that was starved in PAS for 24 h. *A. castellanii* trophozoites were incubated in a 6-well dish with their original medium (100% or 20% PYG) for 30 min, and each well contained 5.0 × 10^5^ trophozoites. After trophozoite attachment, the wells were briefly washed with PAS, and the medium was subsequently replaced with 1 mL PAS containing 5.0 × 10^6^ CFU *E. coli* DH5α (MOI 10). The bacterial concentration was measured by a viable cell count in triplicate.

### RNA-seq

The method used for analysis of RNA expression was described previously [25]. In brief, the total RNA of *A. castellanii* was extracted with Direct-zol™ RNA MiniPrep (ZYMO RESEARCH) according to the manufacturer’s protocol. The samples were treated with DNase I at 37 °C for 30 min. DNase I was removed by phenol‒chloroform extraction, and total RNA was reprecipitated using 95% ethanol. The total RNA was dissolved in nucleic acid-free water. The RNA quality of the control group (concentration: 68 µg/µL, OD260/280: 1.62, OD260/230: 2.00, RIN value: 7.50) and E3 group (concentration: 38 µg/µL, OD260/280: 1.73, OD260/230: 3.17, RIN value: 7.30) was determined. A total amount of 1 µg RNA was used as input material for each sample in RNAseq analysis.

The RNA samples were prepared and sequenced with standard Illumina protocols. As a result, two paired-end (2*150) transcriptome RNAseq datasets (one control and one treatment) were generated. To determine the gene expression profiles of amoebae, the mRNA data (including 28,949 genes) were downloaded from the NCBI database, followed by analysis with CLC Genomics Workbench (version 9.5.4). In summary, the RNAseq data were first loaded into CLC, followed by trimming low-quality sequences with the parameter specified: limit = 0.05. Next, we mapped the reads after trimming to the mRNA dataset to determine the gene expression profile with the default parameters. Finally, CLC conducted analysis to determine the fold change and p value by comparison of the treatment and control. The gene expression values are presented in Table S2.

### Extraction of cellular metabolites

*A. castellanii* trophozoites were lysed with 80% methanol and centrifuged at 12,000 × g for 30 min at 4 ℃ [26]. The supernatant was collected and evaporated by nitrogen. After being vortexed for 1 min and centrifuged at 12,000 × g for 10 min at 4℃, the samples were reconstituted with 100 µL of 0.1 N HCl. After centrifugation, 16 µL of the supernatant was mixed with 4 µL of internal standard (norvaline 250 µM) [26] and 60 µL of working buffer (borate buffer, pH 8.8). Derivatization was initiated by adding 20 µL of 10 mM AQC (6-aminoquinoly N-hydroxysuccinimidyl carbamate obtained from Waters Corporation, Milford, MA, USA) in acetonitrile [27]. After 10 min of incubation, the reactant was mixed with an equal volume of Eluent A (20 mM ammonium formate/0.6% formic acid/1% acetonitrile) and detected using a Waters ACQUITY UPLC System (Waters Corp., Milford, MA, USA). The AQC derivatization reagent was mixed with mixtures of 22 amino acid standards (histidine, asparagine, aspartate, taurine, serine, glutamine, arginine, glycine, citrulline, glutamate, threonine, alanine, proline, ornithine, lysine, tyrosine, methionine, valine, isoleucine, leucine, phenylalanine, and tryptophan) prepared with different concentrations (0, 25, 50, 100, 250, 500 µM) for each amino acid and analyzed by the same procedure.

### Metabolomic and pathway analyses

The targeted amino acid metabolomics method (Biotools Co., Ltd, Taiwan) used was designed to investigate 22 amino acids and to analyze the amino acid composition of each amoeba group. The composition analysis of cellular amino acids (heatmap, volcano plot, and principal components analysis) was performed by the web-based tool MetaboAnalyst 5.0 with one-factor statistical analysis [28]. The pathway maps combining metabolomics and RNA-seq were analyzed by the web-based tool Pathview [29–31] and Kyoto Encyclopedia of Genes and Genomes (KEGG) [32–34].

### Growth curve for amoeba with long-term exposure

To investigate the growth of amoebas with long-term exposure to coculture conditions, the growth curves of the following new groups of amoebas were compared: (1) the LN-ctrl group, amoebas cultured with 20% PYG medium without heat-killed bacteria, and (2) the HKB group, amoebas cultured with 20% PYG medium with 2.0 × 10^6^ CFU heat-killed bacteria. The medium was replaced every 2 days. To obtain the long-term growth curve, the amoebae were harvested every 2 days and counted. Then, the amoebae were collected by centrifugation, and the pellet was resuspended in new medium. A total of 100 µg/mL ampicillin was added to each medium in short and long exposure settings.

### Statistical analysis

GraphPad Prism software (version 7.0; La Jolla, CA, USA) was applied to accomplish the statistical analysis. Data are presented as the mean ± standard deviation (SD). Two-way ANOVA was applied to determine the significance in the bacterial counting experiment.

## Results

### Short-term medium exposure

**Fig. 1:**
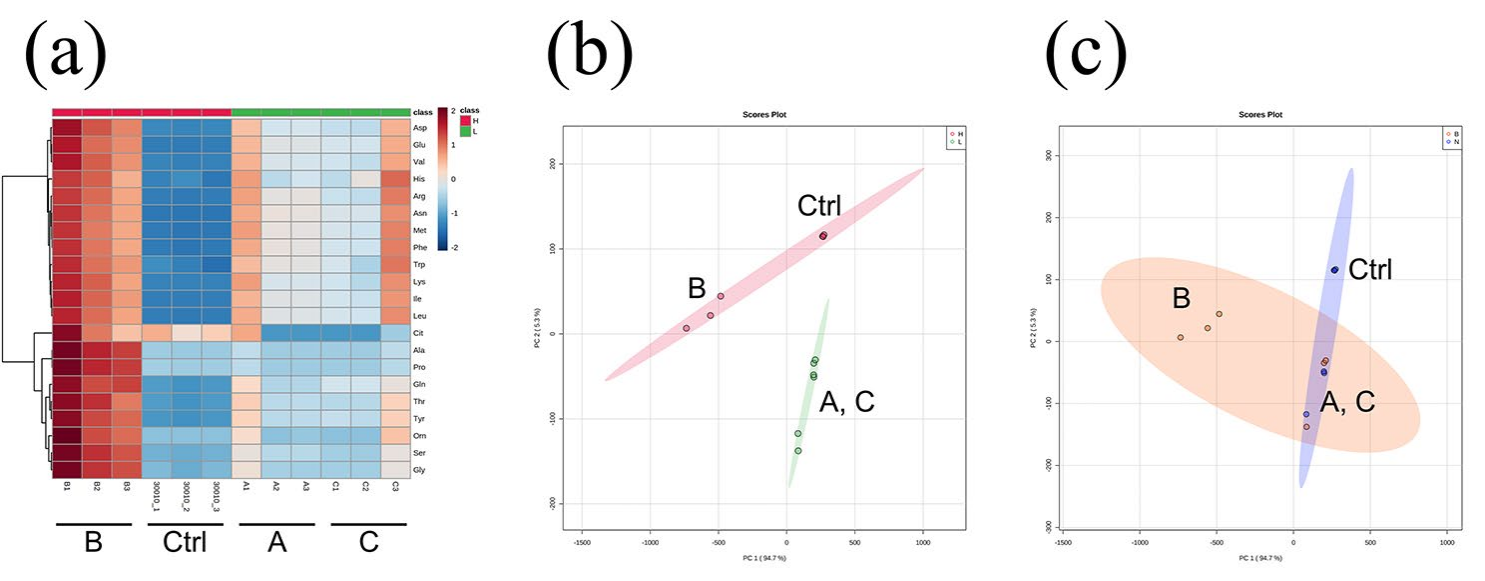
Cellular amino acid composition under short-term exposure. (**a**) Heatmap of amino acid concentrations. (**b**) The 2D PCA result comparing the types of medium; pink: group cultured in normal PYG medium (Control group and B); green: in 20% PYG (Groups A and C). (**c**) The 2D PCA result comparing the presence of heat-killed DH5α; orange: group cultured with heat-killed DH5α (Groups B and C); blue: without heat-killed DH5α (control group and A)

The coculture conditions contained the following factors: diluted low-nutrient medium and heat-killed bacteria. To clarify the effects of these two factors, we designed 3 groups (A, B, and C) for *A. castellanii* samples that were exposed to different media for 3 days of culture treatments. After exposure, the various amino acid concentrations obtained by targeted metabolomic analysis were determined, as presented in Table S1. Compared to the control, the 3 experimental groups (A, B, and C) exhibited higher concentrations of most cellular amino acids except citrulline (Fig. 1a, Table S1). Principal component analysis (PCA) demonstrated that 3-day exposure to two different media (undiluted PYG and 20% diluted PYG medium) caused changes in the intracellular amino acid composition in *A. castellanii*; however, the presence of heat-killed bacteria did not significantly influence the amino acid composition (Fig. 1b, c).

### Reaching amino acid homeostasis after 21 days in the *Acanthamoeba* E3 group

**Fig. 2:**
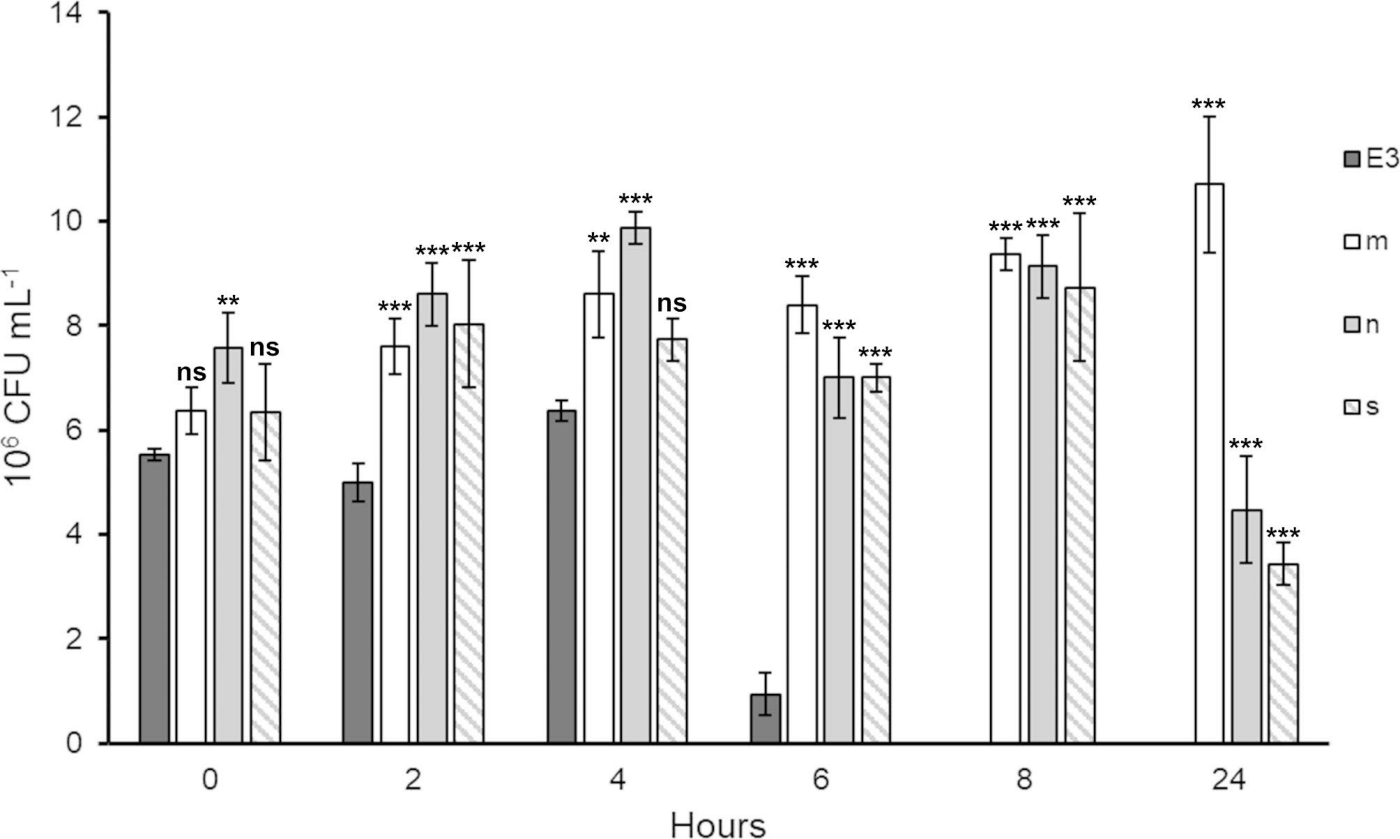
Grazing ability of the long-term exposed amoeba E3 group. The Y-axis is the count of the colony-forming units of the living *E. coli* DH5α remaining in the medium. E3: *E. coli* DH5α cocultured with *Acanthamoeba* E3 group; m: only *E. coli* DH5α in medium; n: *E. coli* DH5α cocultured with normally cultured ATCC_30010; s: *E. coli* DH5α cocultured with 24-hour starved ATCC_30010 strain. The significant differences were determined by two-way ANOVA and are indicated by asterisks (ns: nonsignificant, **: P value < 0.01, ***: P value < 0.001)

The E3 group showed stable growth after 21 days of exposure to a coculture medium (20% PYG with heat-killed bacteria) and demonstrated a significantly stronger ability to graze on living *E. coli* DH5α compared to groups of normally cultured or starved *Acanthamoeba* ATCC_30010 (Fig. 2). The bacteria cocultured with the E3 Group *A. castellanii* were detected after 8 h of coincubation. In contrast, the control Groups n (cocultured with the normally cultured ATCC_30010 strain) and s (cocultured with the starved ATCC_30010 strain) retained 4.47 × 10^6^ and 3.43 × 10^6^ CFU/mL bacteria, respectively.

**Fig. 3:**
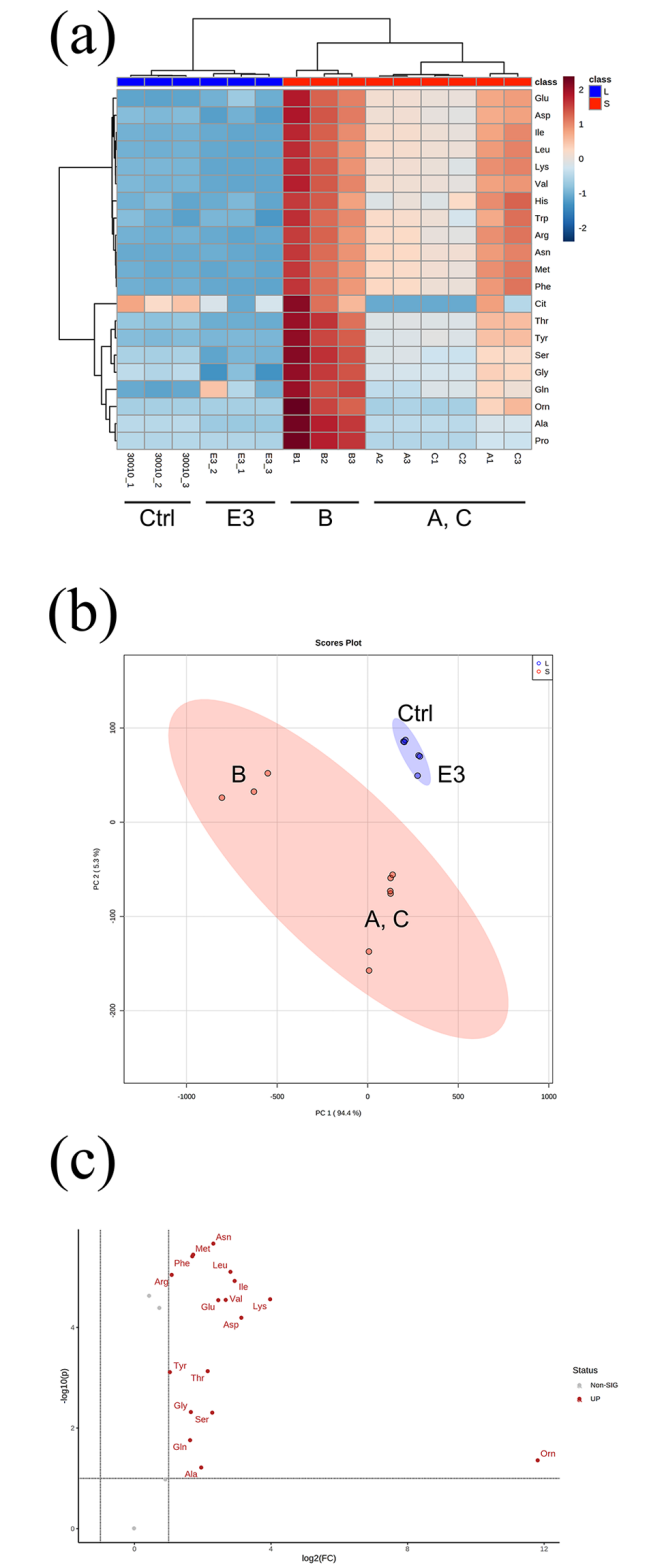
Comparing the amino acid composition of short- and long-term exposed groups. (**a**) Heatmap of amino acid concentrations. (**b**) The 2D PCA result; blue: the long-term stable treated groups (Control and E3); and orange: the short-term treated groups (Groups A, B, C). (**c**) Volcano plot comparing the short-term/long-term fold change of each amino acid. The Y-axis is the p value, and the X-axis is the log_2_-fold change

Similar to the control group, the heatmap demonstrated that the E3 group exhibited a lower concentration of most amino acids than the short-term treated Groups A, B, and C (Fig. 3a, c). Ornithine could not be detected in either the control group or the E3 group. Principal component analysis also indicated that the amino acid composition of the E3 group was more similar to that of the control group (Fig. 3b).

### Amino acid pathways regulated in the E3 group

**Fig. 4:**
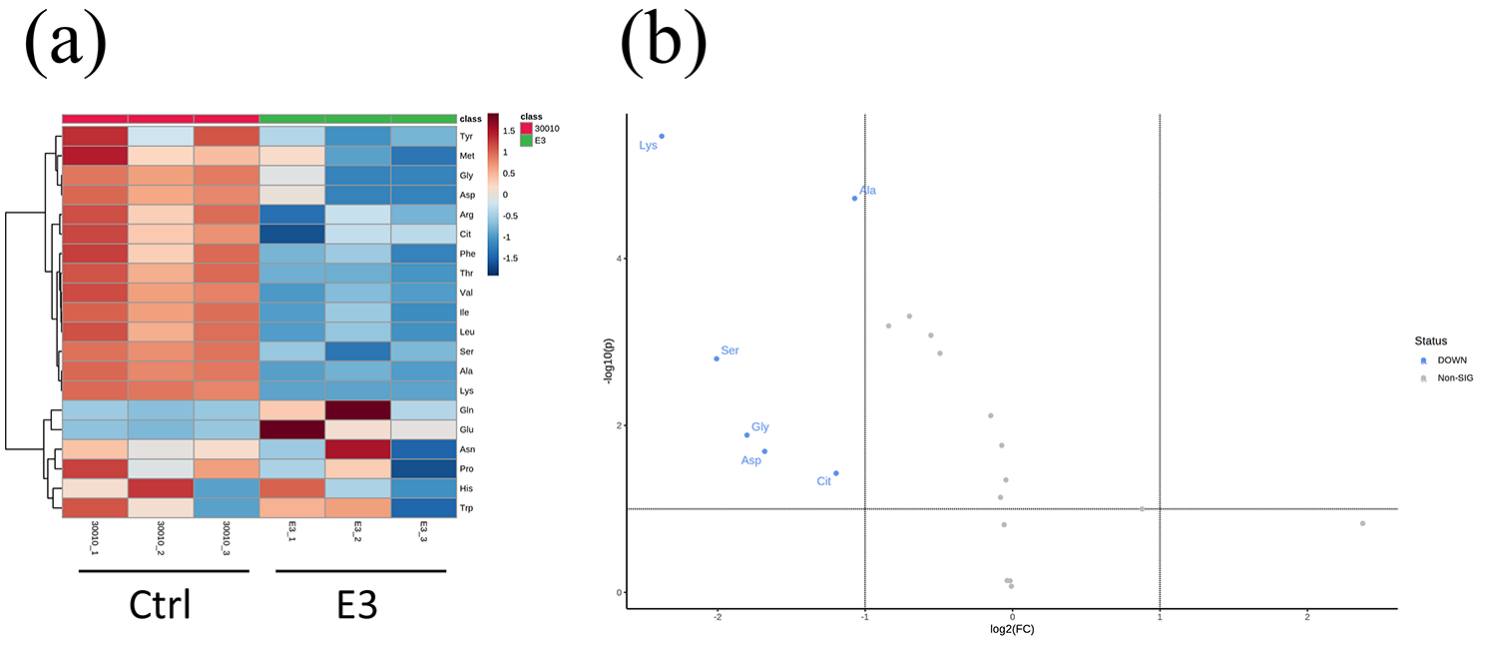
Comparing the amino acid composition of the control and E3 groups. (**a**) Heatmap of amino acid concentrations in the control and E3 groups. (**b**) Volcano plot comparing the E3/Control fold change of each amino acid. The Y-axis is the p value, and the X-axis is the log_2_-fold change

**Fig. 5:**
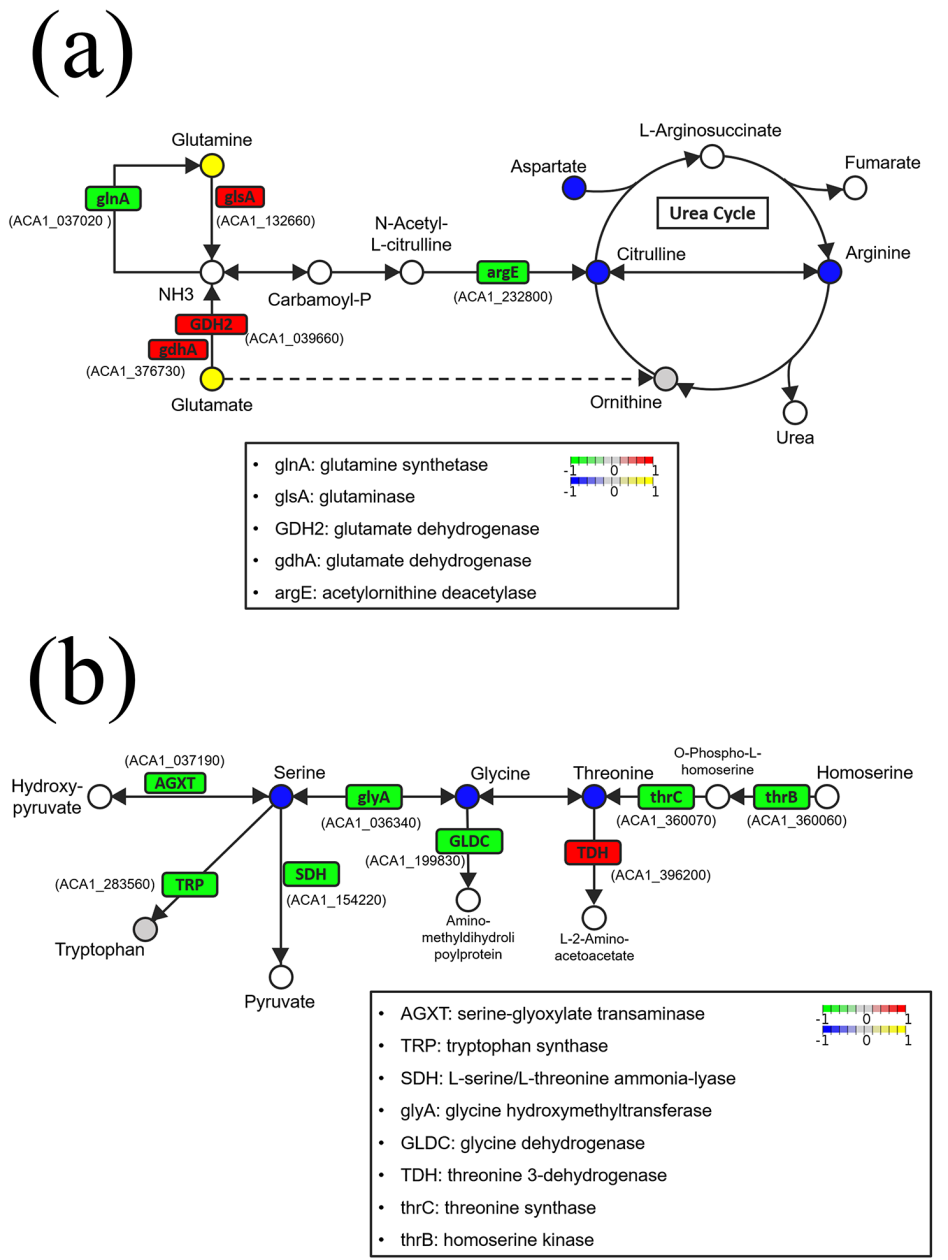
Regulation of amino acid pathways in E3 group amoebae. (**a**) Arginine biosynthesis pathway (KEGG map: 00220). (**b**) Glycine-serine-threonine metabolism pathway (KEGG map: 00260). Metabolites are presented in the round color block, and genes are presented in the square color block. Red indicates upregulated genes, green indicates downregulated genes, yellow indicates enriched metabolites, and blue indicates depleted metabolites. These results were obtained from the KEGG pathway map

To further investigate the regulation of amino acid metabolism in the E3 group, the amino acid compositions were compared between the E3 and control groups (Fig. 4a, Table S1). The E3 group showed a significantly lower concentration of lysine, alanine, serine, glycine, aspartate, and citrulline (see volcano plots Fig. 4b). When combining the metabolomic results and the RNA-seq data, two regulatory pathways seem to be important in the E3 group; in the arginine biosynthesis pathway (KEGG map: 00220), the amino acids that participate in the urea cycle exhibited low concentrations, including aspartate, arginine, and citrulline. Furthermore, the concentrations of glutamine and glutamate were higher, and the expression of the related genes was higher (Fig. 5a). In glycine-serine-threonine metabolism (KEGG map: 00260), these 3 amino acids were present at low concentrations, and most of the related enzymes were downregulated (Fig. 5b).

### Acclimation of *A. castellanii* under long-term coculture conditions

**Fig. 6:**
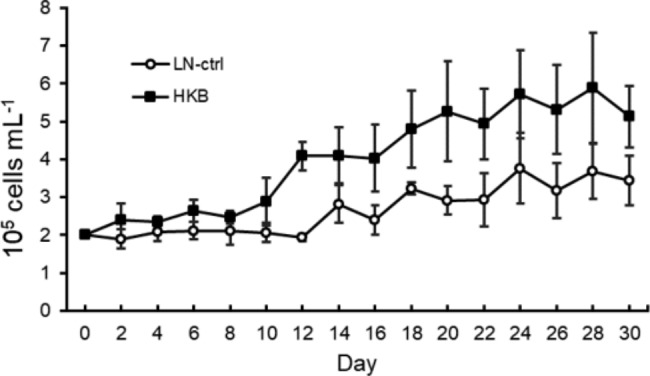
The growth curve in low-nutrient medium with and without heat-killed bacteria. LN-ctrl group: ATCC_30010 strain cultured in 20% PYG medium without heat-killed bacteria, HKB group: ATCC_30010 strain cultured in 20% PYG medium with heat-killed DH5α.

We compared the growth of the ATCC_30010 strain grown in 20% PYG medium with heat-killed DH5α to the growth of the ATCC_30010 strain cultured in 20% PYG medium without heat-killed bacteria. We evaluated both growth curves over 30 days (Fig. 6, HKB and LN-ctrl, respectively). The HKB group started the log phase on Day 10 and presented a temporary stationary phase for 4 days (Days 12–16). After Day 16, the population increased again and showed a similar composition to the LN-ctrl group. Compared to the LN-ctrl group, the HKB group exhibited a larger population.

## Discussion

This study demonstrates that the amino acid composition of *A. castellanii* is sensitive to variations in nutrient environments. Significant differences were observed in the compositions of amino acids between *A. castellanii* cultured in low-nutrient medium (A and C) and high-nutrient medium (Control and B). The presence of heat-killed bacteria (B and C groups) did not seem to influence the amino acid composition.

The short-term exposure groups exhibited greater diversity of cellular amino acids with different compositions. The amino acid level increased more in Group B than in Groups A and C but still showed a similar composition to the control group. Group B was cultured in 100% PYG with heat-killed bacteria. The increase in amino acid levels in the B groups might have been caused by the increase in food sources. On the other hand, the amino acid expression in Groups A and C might have been reprogrammed due to starvation. Eukaryotic cells accumulate the most essential amino acids in response to short-term amino acid starvation [22]. Several factors cause organisms to accumulate amino acids, such as drought stress [35], osmotic stress [36], and growth phase [37]. This study indicates that different amino acid compositions might represent corresponding stimulation (increase or decrease in nutrients), but the mechanisms of amino acid accumulation in *A. castellanii* need to be further studied.

Our study indicates that, under short-term exposure, the type of medium has a more powerful effect on the amino acid composition in amoeba cells than the presence of heat-killed bacteria. However, previous studies found that bacteria could induce significant differences in amoebic growth, metabolism, and encystation [7, 12]. The influence of bacteria on growth, metabolism, and encystation might be caused by the effector proteins secreted by living bacteria. For instance, *Legionella pneumophila* and *Vibrio cholerae* have effective type 3 secretion systems [9, 38]. Our experiments were performed with heat-killed bacteria, which do not secrete effector proteins. However, microbes without secretion systems can also affect amoebic growth, metabolism, and encystation [39]. Therefore, to understand the amoeba-bacteria interaction, changes in the physiology of *A. castellanii* should not be ignored.

To prevent the overgrowth of bacteria when studying amoeba-bacteria interactions, the experimental design included non or low-nutrient media. However, the medium composition influences the intracellular amino acid composition of *A. castellanii*. We aimed to study whether changes in the intracellular amino acid composition were stable under coculture with bacteria. Therefore, *A. castellanii* Group E3 was exposed to 20% PYG medium under coculture conditions with heat-killed bacteria for 21 days. After 21 days of exposure, the amoeba population was morphologically stable when observed under the microscope. Compared to the short-term exposure groups, the E3 group exhibited a more similar amino acid composition to the control group. This indicates that the amino acid composition of the control and E3 groups might be the normal state for *A. castellanii* trophozoites.

A previous study suggested that heat-killed bacteria might exhibit changes in their cell surface and are not representative of living bacteria [13]. The E3 group (cultured for 21 days in a low-nutrient medium) showed a significantly stronger ability to graze on living bacteria compared to *Acanthamoeba*, which had been starved for only 24 h prior to the experiment (Fig. 2). This result indicates that the ability of the amoeba to graze on living bacteria can be trained by exposure to coculture media.

When we repeated the long-term exposure and constructed a 30-day growth curve, *Acanthamoeba* cultured with heat-killed bacteria in a low-nutrient medium showed a biphasic growth pattern. The pattern of the second wave (after 16 days) was similar to that of *Acanthamoeba* grown without heat-killed bacteria. Therefore, the first wave (8–12 days) was influenced by the consumption of heat-killed bacteria, and the second wave reflected acclimation to a low-nutrient environment. In other words, the amoeba constantly grazed on bacteria and acclimated to the low-nutrient environment later during the 30-day culture period. Based on this result, acclimation and bacterial predation seemed to be two independent events. However, Fig. 2 indicates that amoebae have a stronger grazing ability after they acclimate to the environment. To clarify the relationship and mechanisms, further studies are needed.

We further compared the amino acid compositions between the E3 group and the control group. Intracellular levels of lysine, alanine, serine, glycine, aspartate, and citrulline in the E3 group were significantly lower than those in the control group. Based on this combined with RNA-seq data, we conclude that 2 metabolic pathways regulated amino acid homeostasis in the E3 group. This includes the arginine biosynthesis pathway (KEGG map: 00220) and glycine-serine-threonine metabolism (KEGG map: 00260). In the arginine biosynthesis pathway, the E3 group had lower concentrations of aspartate, citrulline, and arginine than the control group. Ornithine could not be detected in either the control or the E3 group. The E3 group contained higher concentrations of glutamine and glutamate. Glutamine metabolism is essential for nitrogen assimilation, detoxification, and general nitrogen metabolism. A previous study compared parasitic and free-living protists and found that free-living protists must exhibit a more responsive nitrogen-handling capability [40]. Glycine-serine-threonine metabolism is important for regulating high-salt stress and the exchange of ions in plants and animals [41, 42]. Although the PYG medium and PAS were isotonic solutions, the increase in phagocytosis of *A. castellanii* might change the ion balance [43–45].

Lysine was significantly decreased in the E3 group (Fig. 4b), but *A. castellanii* lacks several lysine-related genes in the KEGG database. Thus, no conclusions can be made based on the RNA-seq data regarding the lysine-related pathway. According to previous studies, lysine levels and catabolic activity in several organisms are highly related to osmotic and oxidative stress [36, 46]. The main lysine catabolism pathway in higher eukaryotes is the saccharopine pathway, which plays an essential role in homeostasis and the regulation of cellular lysine levels. Similar pathways and enzymes have been found in marine bacteria and are associated with detoxification and oxidative stress [47–49]. The *A. castellanii* genome contains 7 saccharopine-related genes, all of which encode saccharopine dehydrogenase. Only 1 of the 7 saccharopine dehydrogenases was mapped in the KEGG pathway. Saccharopine dehydrogenase (ACA1_399240) has been mapped in lysine biosynthesis and degradation (acan00300, acan00310). However, our RNA-seq results did not indicate that the expression of this gene changed. The effects caused by the low level of lysine in *A. castellanii* need to be further studied.

## Conclusion

This study reveals changes in the composition of the intracellular amino acid in *A. castellanii* under different media and with or without coculture conditions. The amino acid composition can be used to investigate the acclimation of amoebae to different environments. Furthermore, with amino acid metabolomics, we demonstrated that the type of nutrient medium can change the amino acid composition. According to the growth curve, it appears that the amoeba does not acclimate to a low-nutrient environment within 3 days. Bacterial grazing continues during these three early days. Therefore, we believe that previous studies may have overlooked the effects of low-nutrient medium and therefore underestimated the growth of amoebae that graze on bacteria. The metabolic pathways related to nitrogen-handling capability and the exchange of ions were regulated under long-term exposure to a coculture medium. These results provide insight into the physiological activities of amoebae for further studies.

## Electronic supplementary material

Below is the link to the electronic supplementary material.

Supplementary Material 1

Supplementary Material 2

## Data Availability

All data generated or analyzed during this study are included in this published article and its supplementary information files. The RNAseq datasets are available in the Gene Expression Omnibus (GEO) repository, accession number: GSE227112 (https://www.ncbi.nlm.nih.gov/geo/query/acc.cgi?acc=GSE227112).

## Abbreviations

AK: Acanthamoeba keratitis
HK: heat-killed
PAS: Page’s amoeba saline
Ctrl: control group
KEGG: Kyoto Encyclopedia of Genes and Genomes
PYG: peptone-yeast extract-glucose
LB: lysogeny broth
HKB: heat-killed bacteria feed group
LN-ctrl: low-nutrient medium control group
